# Supplementary material for: Annotation of Potential Vaccine Targets and Designing of mRNA-Based Multi-Epitope Vaccine against Lumpy Skin Disease Virus via Reverse Vaccinology and Agent-Based Modeling
Source: Bioengineering (Basel). 2023 Mar 28;10(4):430. doi: 10.3390/bioengineering10040430 (PMC10135540; doi:10.3390/bioengineering10040430)
Supplement: Supplementary file 1 [file bioengineering-10-00430-s001.zip › bioengineering-2233708-supplementary.pdf]

Supplementary Materials:

Table S1: Bovine alleles used for T –cells epitope prediction.

S.NO	MHC1	MHCII
01	BoLA-T2C	BoLA-DRB3*0101
02	BoLA-HD6	BoLA-DRB3*0401
03	BoLA-D18.4	BoLA-DRB3*0501
04	BoLA-T2a	BoLA-DRB3*0801
05	BoLA-T2b	BoLA-DRB3*1103
06	BoLA-JSP.1	BoLA-DRB3*1902

Table S2: Designed vaccine constructs with allergenicity, antigenicity and solubility analysis.

**Vaccine construct**	**Adjuvant**	**Construct**	**Allergenicity**	**Antigenpro**	**Vaxijen**	**Solubility Protein-sol**	Dock TLR2	Dock TLR4
LSDV-V1	Beta defensin adjuvant	EAAAKLALLFLVLSAGSGFTQGVRNHVTCRINRGFCVPIRCPGRTRQIGTCFGPRIKCCRSWEAAAKAKFVAAWTLKAAAGGGSEGVYLCSITTDTRCNPGPGPGKKALIIKEVKRKYLAAYNVLDYDRSKGGGSHEYGAEALERAGGGGSYGLVKKKNNIWVDVNSGPGPGKKNVSIRHLKVISLAAYAIFMLVSTIGGGSHEYGAEALERAGGGGSYTTQQYCNVSPFINDNGPGPGKKKTDLSLLKRRIQAAYDKKGCIVEFHEYGAEALERAGAKFVAAWTLKAAAGGGS	Non allergen-Allertop	0.792602	0.5682	0.663	-884.2	-963.8
LSDV - V2	Beta defensin adjuvant	EAAAKLALLFLVLSAGSGFTQGVRNHVTCRINRGFCVPIRCPGRTRQIGTCFGPRIKCCRSWEAAAKAKFVAAWTLKAAAGGGSNSVIGTNYELLCINTKGPGPGKKLIIKEVKRKYLSAAYNSTIALGKNGGGSHEYGAEALERAGGGGSLSNIKKSSKGDINACYGPGPGKKVSYIICVKRLYNAAYNVSCNYVSYGGGSHEYGAEALERAGGGGSKGCIVEFGSQEKVCVTGPGPGKKDLSLLKRRIQKVAAYDFWIKFISIHEYGAEALERAGAKFVAAWTLKAAAGGGS	Non allergen-Allertop	0.732347	0.5099	0.636	-796.4	-1157.4

LSDV - V3	Beta defensin adjuvan t	EAAAKLALLFLVLSAGSGFT<br>QGVRNHVTCRINRGFCVPIR<br>CPGRTRQIGTCFGPRIKCCRS<br>WEAAAKAKFVAAWTLKAA<br>AGGGSSITTDTRCNPKNLAL<br>KGPGPGKKIALIIKEVKRKYA<br>AYTVNFLNSTIGGGSHEYGA<br>EALERAGGGGSSCNYVSYIIC<br>VKRLYNGPGPGKKSIRHLKV<br>ISLTYAAYNYVSYIICVGGGS<br>HEYGAEALERAGGGGSKGCI<br>VEFGSQEKVCVTGPGPGKKV<br>FIKRQDVNTVLAAYNTDDF<br>WIKFHEYGAEALERAGAKFV<br>AAWTLKAAAGGGS	Non allergen-Allertop	0.779016	0.5364	0.625	-858.1	-1026.2

Table S3: Refine models of vaccine construct LSDV-V2 predicted by GalaxyRefine server

Model	GDT-HA	RMSD	MolProbity	Clash score	Poor rotamers	Rama favored
Initial	1.0000	0.000	1.846	5.4	0.0	89.7
MODEL 1	0.9872	0.290	2.097	12.4	0.9	91.8
MODEL 2	0.9906	0.264	2.157	15.3	0.9	92.5
MODEL 3	0.9881	0.279	2.176	15.1	0.5	91.8
MODEL 4	0.9855	0.298	2.192	14.8	0.5	91.1
MODEL 5	0.9847	0.307	2.224	17.5	0.0	92.1

Table S4: DNA and RNA sequences of the peptide vaccine constructs.

**Vaccine construc t**	**Adjuva nt**	**Construct**	**DNA Sequences**	**RNA Sequences**
LSDV-V1	Beta defensin adjuvan t	EAAAKLALLFLVLSAGSGFTQ<br>GVRNHVTCRINRGFCVPIRCP<br>GRTRQIGTCFGPRIKCCRSWE<br>AAAKAKFVAAWTLKAAAGG<br>GSEGVYLCSITTDTRCNPGPG<br>PGKKALIIKEVKRKYLAAYNV<br>LDYDRSKGGGSHEYGAEALE<br>RAGGGGSYGLVKKKNNIWV<br>DVNSGPGPGKKNVSIRHLKVI<br>SLAAYAIFMLVSTIGGGSHEY<br>GAEALERAGGGGSYTTQQYC<br>NVSPFINDNGPGPGKKKTDLS<br>LLKRRIQAAYDKKGCIVEFHE	GAAGCTGCTGCTAAACT<br>GGCTCTGCTGTTCCTGGT<br>TCTGTCTGCTGGTTCTGG<br>TTTCACCCAGGGTGTTC<br>GTAACCACGTTACCTGC<br>CGTATCAACCGTGGTTT<br>CTGCGTTCCGATCCGTT<br>GCCCGGGTCGTACCCGT<br>CAGATCGGTACCTGCTT<br>CGGTCCGCGTATCAAAT<br>GCTGCCGTTCTTGGGAA<br>GCTGCTGCTAAAGCTAA<br>ATTCGTTGCTGCTTGGAC	GAAGCUGCUGCUAAACU<br>GGCUCUGCUGUUCCUGG<br>UUCUGUCUGCUGGUUCU<br>GGUUUCACCCAGGGUGU<br>UCGUAACCACGUUACCU<br>GCCGUAUCAACCGUGGU<br>UUCUGCGUUCCGAUCCG<br>UUGCCCGGGUCGUACCC<br>GUCAGAUCGGUACCUGC<br>UUCGGUCCGCGUAUCAA<br>AUGCUGCCGUUCUUGGG<br>AAGCUGCUGCUAAAGCU<br>AAAUUCGUUGCUGCUUG

		YGAEALERAGAKFVAAWTLK<br>AAAGGGS	CCTGAAAGCTGCTGCTG<br>GTGGTGGTTCTGAAGGT<br>GTTTACCTGTGCTCTATC<br>ACCACCGACACCCGTTG<br>CAACCCGGGTCCGGGTC<br>CGGGTAAAAAAGCTCTG<br>ATCATCAAAGAAGTTAA<br>ACGTAAATACCTGGCTG<br>CTTACAACGTTCTGGAC<br>TACGACCGTTCTAAAGG<br>TGGTGGTTCTCACGAAT<br>ACGGTGCTGAAGCTCTG<br>GAACGTGCTGGTGGTGG<br>TGGTTCTTACGGTCTGGT<br>TAAAAAAAAAAACAAC<br>ATCTGGGTTGACGTTAA<br>CTCTGGTCCGGGTCCGG<br>GTAAAAAAAACGTTTCT<br>ATCCGTCACCTGAAAGT<br>TATCTCTCTGGCTGCTTA<br>CGCTATCTTCATGCTGGT<br>TTCTACCATCGGTGGTG<br>GTTCTCACGAATACGGT<br>GCTGAAGCTCTGGAACG<br>TGCTGGTGGTGGTGGTT<br>CTTACACCACCCAGCAG<br>TACTGCAACGTTTCTCC<br>GTTCATCAACGACAACG<br>GTCCGGGTCCGGGTAAA<br>AAAAAAACCGACCTGTC<br>TCTGCTGAAACGTCGTA<br>TCCAGGCTGCTTACGAC<br>AAAAAAGGTTGCATCGT<br>TGAATTCCACGAATACG<br>GTGCTGAAGCTCTGGAA<br>CGTGCTGGTGCTAAATT<br>CGTTGCTGCTTGGACCC<br>TGAAAGCTGCTGCTGGT<br>GGTGGTTCT	GACCCUGAAAGCUGCUG<br>CUGGUGGUGGUUCUGA<br>AGGUGUUUACCUGUGCU<br>CUAUCACCACCGACACC<br>CGUUGCAACCCGGGUCC<br>GGGUCCGGGUAAAAAA<br>GCUCUGAUCAUCAAAGA<br>AGUUAAACGUAAAUAC<br>CUGGCUGCUUACAACGU<br>UCUGGACUACGACCGUU<br>CUAAAGGUGGUGGUUC<br>UCACGAAUACGGUGCUG<br>AAGCUCUGGAACGUGCU<br>GGUGGUGGUGGUUCUU<br>ACGGUCUGGUUAAAAA<br>AAAAAACAACAUCUGG<br>GUUGACGUUAACUCUGG<br>UCCGGGUCCGGGUAAAA<br>AAAACGUUUCUAUCCGU<br>CACCUGAAAGUUAUCUC<br>UCUGGCUGCUUACGCUA<br>UCUUCAUGCUGGUUUCU<br>ACCAUCGGUGGUGGUUC<br>UCACGAAUACGGUGCUG<br>AAGCUCUGGAACGUGCU<br>GGUGGUGGUGGUUCUU<br>ACACCACCCAGCAGUAC<br>UGCAACGUUUCUCCGUU<br>CAUCAACGACAACGGUC<br>CGGGUCCGGGUAAAAAA<br>AAAACCGACCUGUCUCU<br>GCUGAAACGUCGUAUCC<br>AGGCUGCUUACGACAAA<br>AAAGGUUGCAUCGUUG<br>AAUUCCACGAAUACGGU<br>GCUGAAGCUCUGGAACG<br>UGCUGGUGCUAAAUUCG<br>UUGCUGCUUGGACCCUG<br>AAAGCUGCUGCUGGUGG<br>UGGUUCU

LSDV - V2	Beta defensin adjuvant	EAAAKLALLFLVLSAGSGFTQ<br>GVRNHVTCRINRGFCVPIRCP<br>GRTRQIGTCFGPRIKCCRSWE<br>AAAKAKFVAAWTLKAAAGG<br>GSNSVIGTNYELLCINTKGPG<br>PGKKLIIKEVKRKYLSAAYNST<br>IALGKNGGGSHEYGAEALER<br>AGGGGSLSNIKKSSKGDINAC<br>YGPGPGKKVSYIICVKRLYNA<br>AYNVSCNYVSYGGGSHEYGA<br>EALERAGGGGSKGCIVEFGSQ<br>EKVCVTGPGPGKKDLSLLKRR<br>IQKVAAYDFWIKFISIHEYGAE<br>ALERAGAKFVAAWTLKAAA<br>GGGS	GAAGCTGCTGCTAAACT<br>GGCTCTGCTGTTCCTGGT<br>TCTGTCTGCTGGTTCTGG<br>TTTCACCCAGGGTGTTC<br>GTAACCACGTTACCTGC<br>CGTATCAACCGTGGTTT<br>CTGCGTTCCGATCCGTT<br>GCCCGGGTCGTACCCGT<br>CAGATCGGTACCTGCTT<br>CGGTCCGCGTATCAAAT<br>GCTGCCGTTCTTGGGAA<br>GCTGCTGCTAAAGCTAA<br>ATTCGTTGCTGCTTGGAC<br>CCTGAAAGCTGCTGCTG<br>GTGGTGGTTCTAACTCT<br>GTTATCGGTACCAACTA<br>CGAACTGCTGTGCATCA<br>ACACCAAAGGTCCGGGT<br>CCGGGTAAAAAACTGAT<br>CATCAAAGAAGTTAAAC<br>GTAAATACCTGTCTGCT<br>GCTTACAACTCTACCAT<br>CGCTCTGGGTAAAAACG<br>GTGGTGGTTCTCACGAA<br>TACGGTGCTGAAGCTCT<br>GGAACGTGCTGGTGGTG<br>GTGGTTCTCTGTCTAACA<br>TCAAAAAATCTTCTAAA<br>GGTGACATCAACGCTTG<br>CTACGGTCCGGGTCCGG<br>GTAAAAAAGTTTCTTAC<br>ATCATCTGCGTTAAACG<br>TCTGTACAACGCTGCTT<br>ACAACGTTTCTTGCAAC<br>TACGTTTCTTACGGTGGT<br>GGTTCTCACGAATACGG<br>TGCTGAAGCTCTGGAAC<br>GTGCTGGTGGTGGTGGT<br>TCTAAAGGTTGCATCGT<br>TGAATTCGGTTCTCAGG<br>AAAAAGTTTGCGTTACC<br>GGTCCGGGTCCGGGTAA<br>AAAAGACCTGTCTCTGC<br>TGAAACGTCGTATCCAG<br>AAAGTTGCTGCTTACGA<br>CTTCTGGATCAAATTCA<br>TCTCTATCCACGAATAC<br>GGTGCTGAAGCTCTGGA<br>ACGTGCTGGTGCTAAAT	GAAGCUGCUGCUAAACU<br>GGCUCUGCUGUUCCUGG<br>UUCUGUCUGCUGGUUCU<br>GGUUUCACCCAGGGUGU<br>UCGUAACCACGUUACCU<br>GCCGUAUCAACCGUGGU<br>UUCUGCGUUCCGAUCCG<br>UUGCCCGGGUCGUACCC<br>GUCAGAUCGGUACCUGC<br>UUCGGUCCGCGUAUCAA<br>AUGCUGCCGUUCUUGGG<br>AAGCUGCUGCUAAAGCU<br>AAAUUCGUUGCUGCUUG<br>GACCCUGAAAGCUGCUG<br>CUGGUGGUGGUUCUAAC<br>UCUGUUAUCGGUACCAA<br>CUACGAACUGCUGUGCA<br>UCAACACCAAAGGUCCG<br>GGUCCGGGUAAAAAACU<br>GAUCAUCAAAGAAGUU<br>AAACGUAAAUACCUGUC<br>UGCUGCUUACAACUCUA<br>CCAUCGCUCUGGGUAAA<br>AACGGUGGUGGUUCUCA<br>CGAAUACGGUGCUGAAG<br>CUCUGGAACGUGCUGGU<br>GGUGGUGGUUCUCUGUC<br>UAACAUCAAAAAAUCU<br>UCUAAAGGUGACAUCA<br>ACGCUUGCUACGGUCCG<br>GGUCCGGGUAAAAAAG<br>UUUCUUACAUCAUCUGC<br>GUUAAACGUCUGUACA<br>ACGCUGCUUACAACGUU<br>UCUUGCAACUACGUUUC<br>UUACGGUGGUGGUUCUC<br>ACGAAUACGGUGCUGAA<br>GCUCUGGAACGUGCUGG<br>UGGUGGUGGUUCUAAA<br>GGUUGCAUCGUUGAAU<br>UCGGUUCUCAGGAAAAA<br>GUUUGCGUUACCGGUCC<br>GGGUCCGGGUAAAAAA<br>GACCUGUCUCUGCUGAA<br>ACGUCGUAUCCAGAAAG<br>UUGCUGCUUACGACUUC<br>UGGAUCAAAUUCAUCUC<br>UAUCCACGAAUACGGUG<br>CUGAAGCUCUGGAACGU

			TCGTTGCTGCTTGGACC CTGAAAGCTGCTGCTGG TGGTGGTTCT	GCUGGUGCUAAAUUCGU UGCUGCUUGGACCCUGA AAGCUGCUGCUGGUGGU GGUUCU
LSDV - V3	Beta defensin adjuvant	EAAAKLALLFLVLSAGSGFTQ GVRNHVTCRINRGFCVPIRCP GRTRQIGTCFGPRIKCCRSWE AAAKAKFVAAWTLKAAAGG GSSITTDTRCNPKNLALKGPG PGKKIALIIKEVKRKYAAYTV NFLNSTIGGGSHEYGAEALER AGGGGSSCNYVSYIICVKRLY NGPGPGKKSIRHLKVISLTYA AYNYVSYIICVGGGSHEYGAE ALERAGGGGSKGCIVEFGSQE KVCVTGPGPGKKVFIKRQDV NTVLAAYNTDDFWIKFHEYG AEALERAGAKFVAAWTLKA AAGGGS	GAAGCTGCTGCTAAACT GGCTCTGCTGTTCCTGGT TCTGTCTGCTGGTTCTGG TTTCACCCAGGGTGTTC GTAACCACGTTACCTGC CGTATCAACCGTGGTTT CTGCGTTCCGATCCGTT GCCCGGGTCGTACCCGT CAGATCGGTACCTGCTT CGGTCCGCGTATCAAAT GCTGCCGTTCTTGGGAA GCTGCTGCTAAAGCTAA ATTCGTTGCTGCTTGGAC CCTGAAAGCTGCTGCTG GTGGTGGTTCTTCTATCA CCACCGACACCCGTTGC AACCCGAAAAACCTGG CTCTGAAAGGTCCGGGT CCGGGTAAAAAAATCG CTCTGATCATCAAAGAA GTTAAACGTAAATACGC TGCTTACACCGTTAACTT CCTGAACTCTACCATCG GTGGTGGTTCTCACGAA TACGGTGCTGAAGCTCT GGAACGTGCTGGTGGTG GTGGTTCTTCTTGCAACT ACGTTTCTTACATCATCT GCGTTAAACGTCTGTAC AACGGTCCGGGTCCGGG TAAAAAATCTATCCGTC ACCTGAAAGTTATCTCT CTGACCTACGCTGCTTA CAACTACGTTTCTTACAT CATCTGCGTTGGTGGTG GTTCTCACGAATACGGT GCTGAAGCTCTGGAACG TGCTGGTGGTGGTGGTT	GAAGCUGCUGCUAAACU GGCUCUGCUGUUCCUGG UUCUGUCUGCUGGUUCU GGUUUCACCCAGGGUGU UCGUAACCACGUUACCU GCCGUAUCAACCGUGGU UUCUGCGUUCCGAUCCG UUGCCCGGGUCGUACCC GUCAGAUCGGUACCUGC UUCGGUCCGCGUAUCAA AUGCUGCCGUUCUUGGG AAGCUGCUGCUAAAGCU AAAUUCGUUGCUGCUUG GACCCUGAAAGCUGCUG CUGGUGGUGGUUCUUCU AUCACCACCGACACCCG UUGCAACCCGAAAAACC UGGCUCUGAAAGGUCCG GGUCCGGGUAAAAAAA UCGCUCUGAUCAUCAAA GAAGUUAAACGUAAAU ACGCUGCUUACACCGUU AACUUCCUGAACUCUAC CAUCGGUGGUGGUUCUC ACGAAUACGGUGCUGAA GCUCUGGAACGUGCUGG UGGUGGUGGUUCUUCU UGCAACUACGUUUCUUA CAUCAUCUGCGUUAAAC GUCUGUACAACGGUCCG GGUCCGGGUAAAAAAUC UAUCCGUCACCUGAAAG UUAUCUCUCUGACCUAC GCUGCUUACAACUACGU UUCUUACAUCAUCUGCG UUGGUGGUGGUUCUCAC GAAUACGGUGCUGAAGC UCUGGAACGUGCUGGUG

			CTAAAGGTTGCATCGTT<br>GAATTCGGTTCTCAGGA<br>AAAAGTTTGCGTTACCG<br>GTCCGGGTCCGGGTAAA<br>AAAGTTTTCATCAAACG<br>TCAGGACGTTAACACCG<br>TTCTGGCTGCTTACAAC<br>ACCGACGACTTCTGGAT<br>CAAATTCCACGAATACG<br>GTGCTGAAGCTCTGGAA<br>CGTGCTGGTGCTAAATT<br>CGTTGCTGCTTGGACCC<br>TGAAAGCTGCTGCTGGT<br>GGTGGTTCT	GUGGUGGUUCUAAAGG<br>UUGCAUCGUUGAAUUCG<br>GUUCUCAGGAAAAAGU<br>UUGCGUUACCGGUCCGG<br>GUCCGGGUAAAAAAGU<br>UUUCAUCAAACGUCAGG<br>ACGUUAACACCGUUCUG<br>GCUGCUUACAACACCGA<br>CGACUUCUGGAUCAAAU<br>UCCACGAAUACGGUGCU<br>GAAGCUCUGGAACGUGC<br>UGGUGCUAAAUUCGUU<br>GCUGCUUGGACCCUGAA<br>AGCUGCUGCUGGUGGUG<br>GUUCU

Table S5: Different residues of vaccine construct and TLR4 involved in hydrogen bonding.

NO. NAME NO. CHAIN NAME NO. CHAIN
1. GLN 39 A <--> GLY 16 B
2. ASP 84 A <--> ARG 46 B
3. ASP 84 A <--> ARG 46 B
4 SER 86 A <--> ARG 46 B
5. ARG 87 A <--> ASN 190 B
6. LYS 130 A <--> GLN 47 B
7. GLU 135 A <--> TRP 62 B
8. GLU 135 A <--> ARG 44 B
9. GLU 135 A <--> ARG 44 B
10. GLU 154 A <--> ARG 40 B
11. ASP 181 A <--> ARG 40 B
12. ASP 181 A <--> ARG 40 B
13. ASP 181 A <--> GLY 43 B
14. ARG 234 A <--> CYS 36 B
15. ARG 257 A <--> PRO 38 B
16. ARG 257 A <--> ILE 39 B
17. LYS 264 A <--> ARG 33 B
18. LYS 264 A <--> GLY 34 B
19. ARG 382 A <--> GLY 133 B
20. ARG 382 A <--> TYR 138 B

21. ASP 617 A <--> ARG 115 B

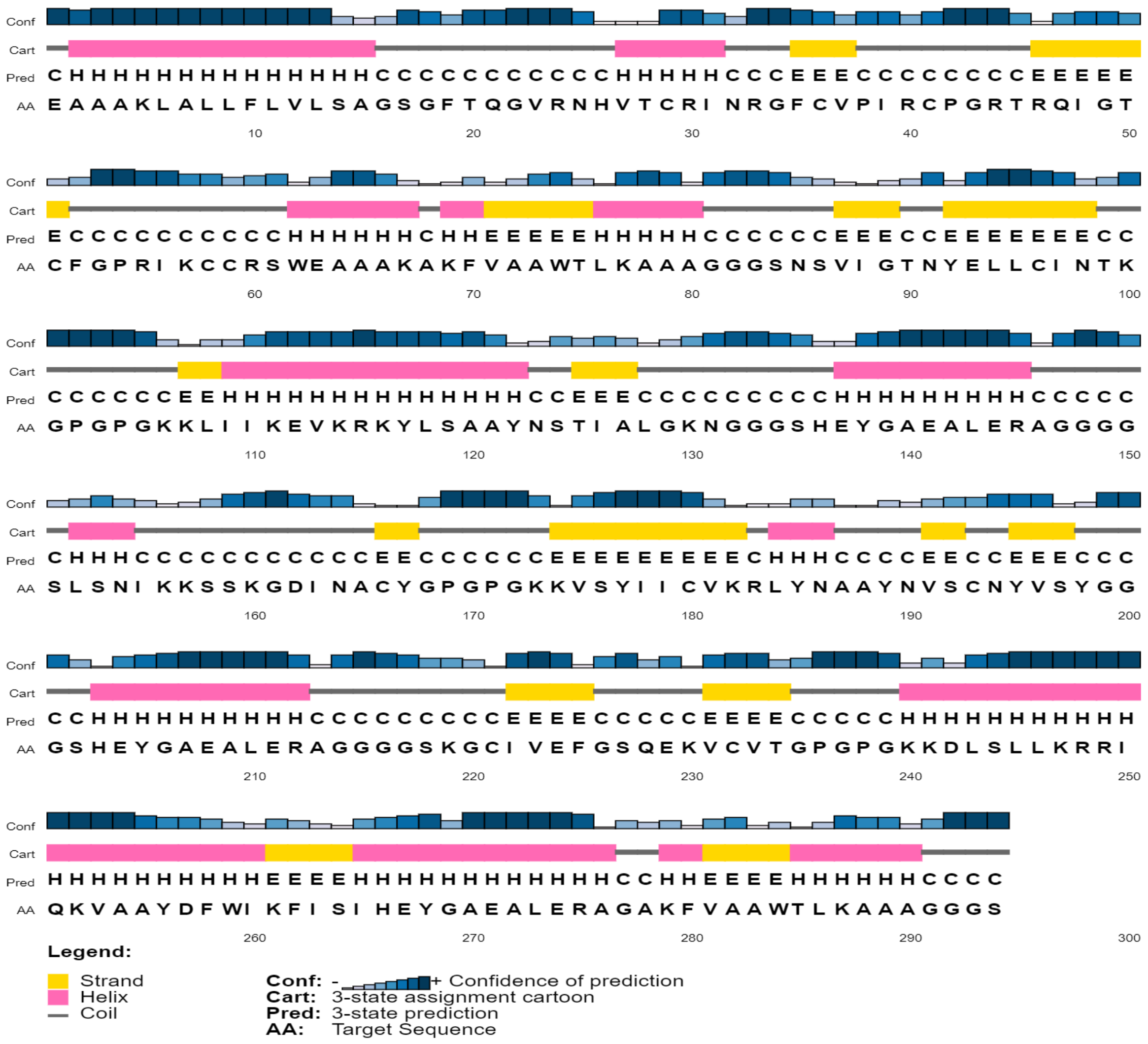

Figure S1a: Secondary structure prediction of LSDV-V1 by PSIPRED server.

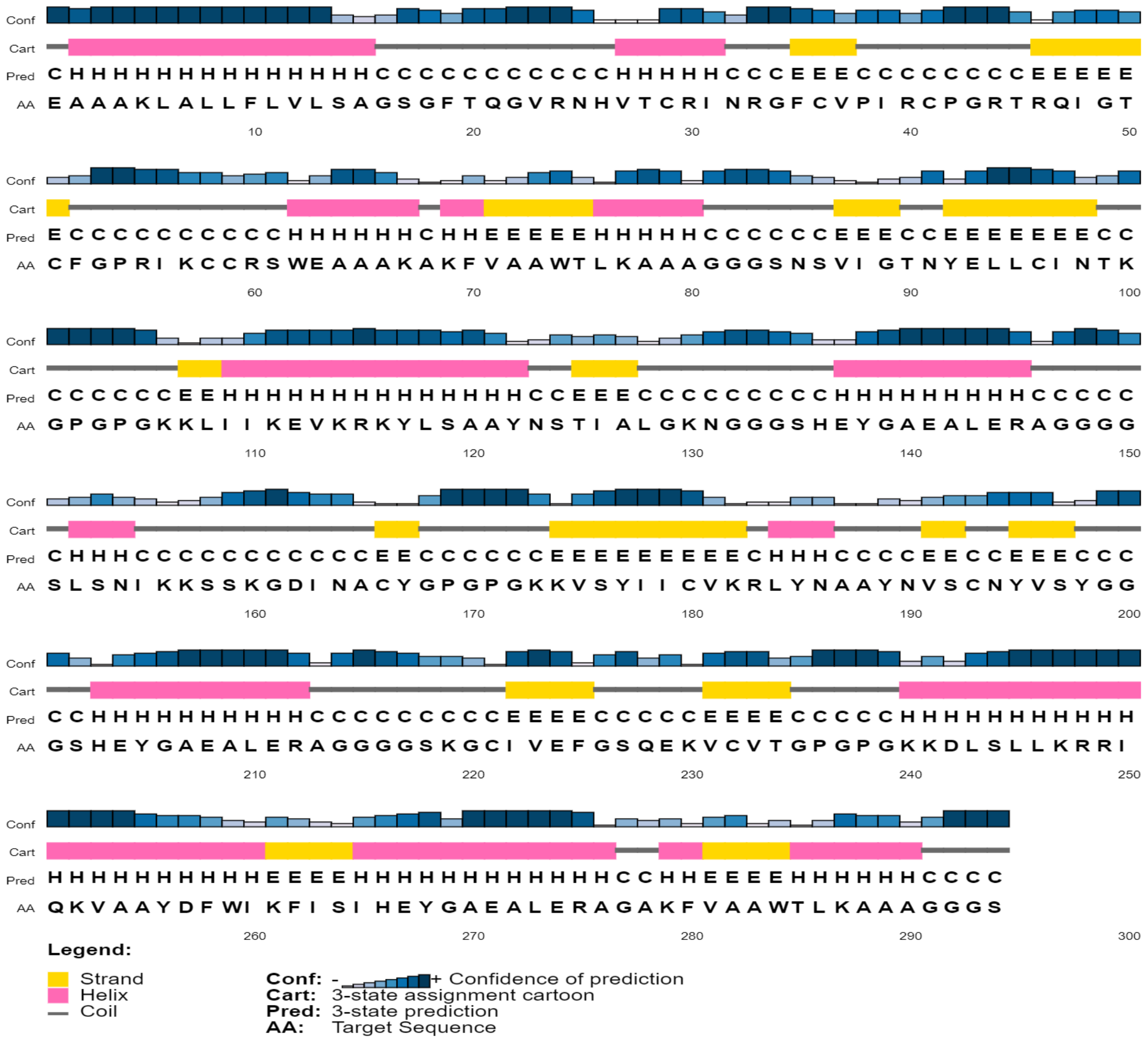

Figure S1b: Secondary structure prediction of LSDV-V2 by PSIPRED server.

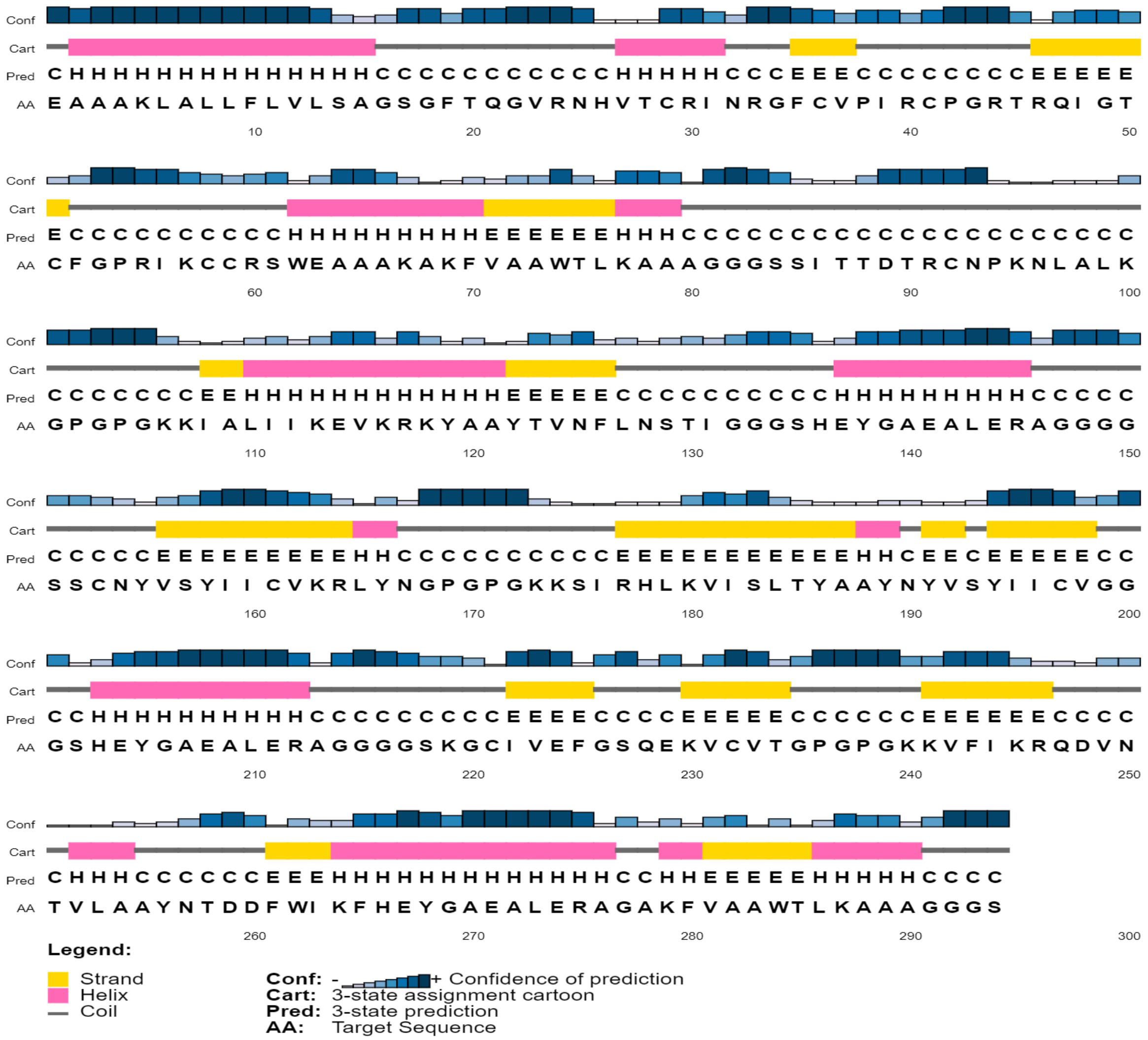

Figure S1c: Secondary structure prediction of LSDV-V3 by PSIPRED server.

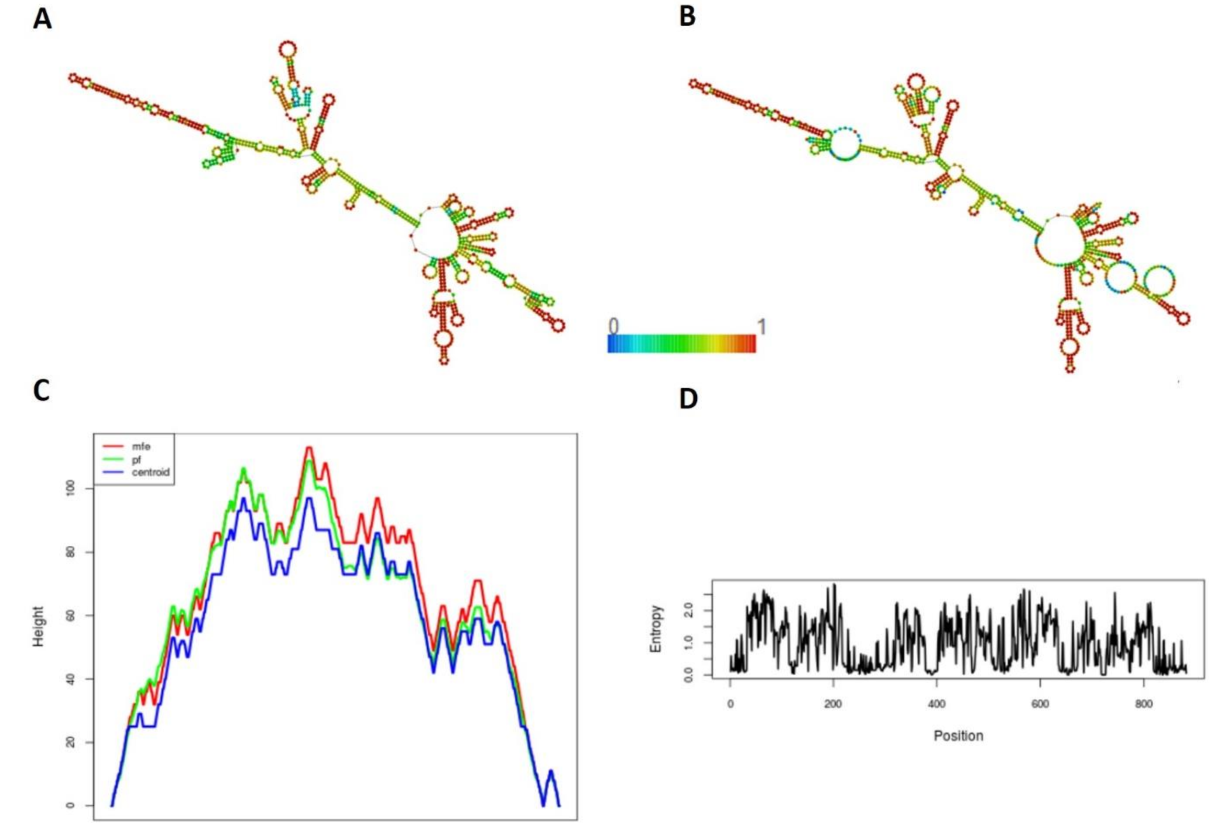

Figure S2a: Secondary Structure prediction of the prioritized LSDV-V1 mRNA construct. (A) Model presentation of minimum free energy of the LSDV-V1 mRNA structure (B) The proposed model of the minimum free energy (MFE) of secondary centroid LSDV-V1 mRNA structure (C) The mountain plot representation of the MFE structure, the thermodynamic ensemble of RNA structures, and the centroid structure. (D) The positional entropy plot for each position.

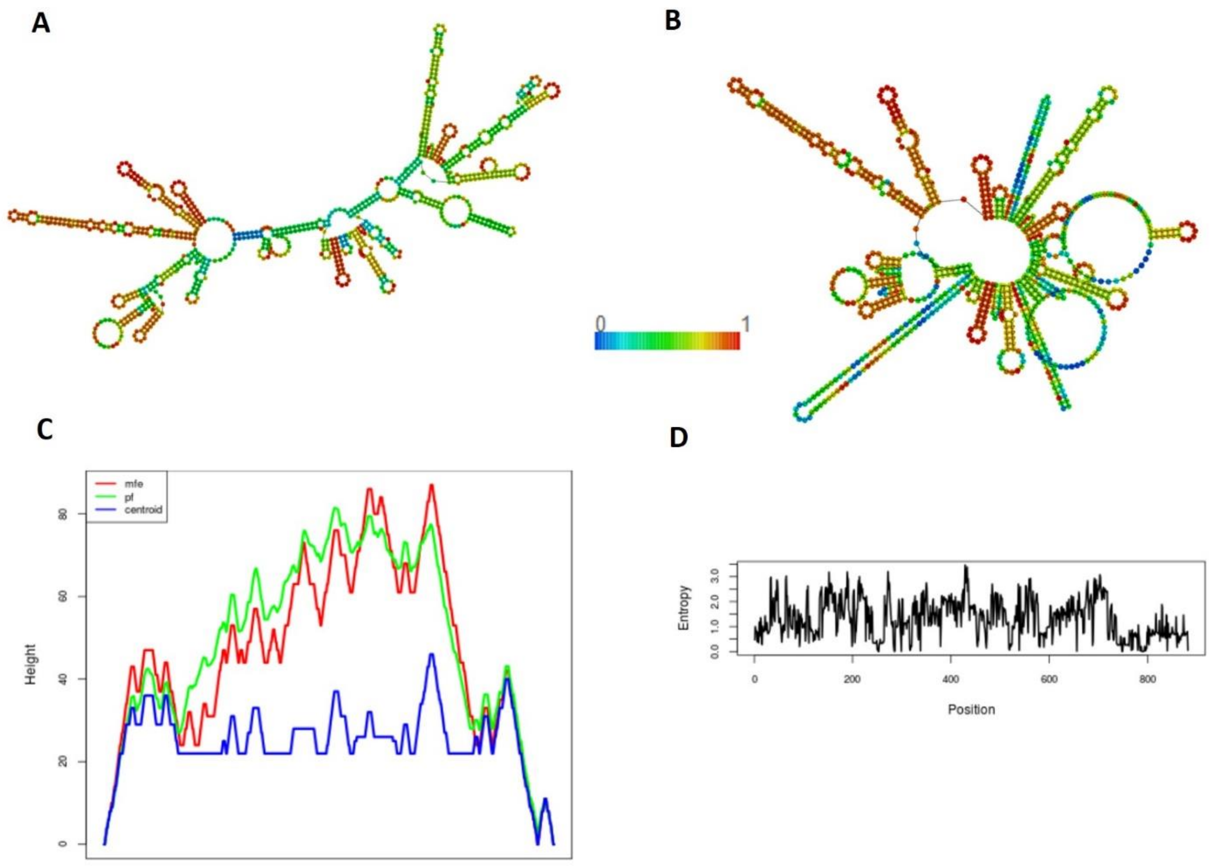

Figure S2b: Secondary Structure prediction of the prioritized LSDV-V3 mRNA construct. (A) Model presentation of minimum free energy of the LSDV-V3 mRNA structure (B) The proposed model of the minimum free energy of secondary centroid LSDV-V3 mRNA structure (C) The mountain plot representation of the MFE structure, the thermodynamic ensemble of mRNA structure, and the centroid structure. (D) The positional entropy plot for each position.

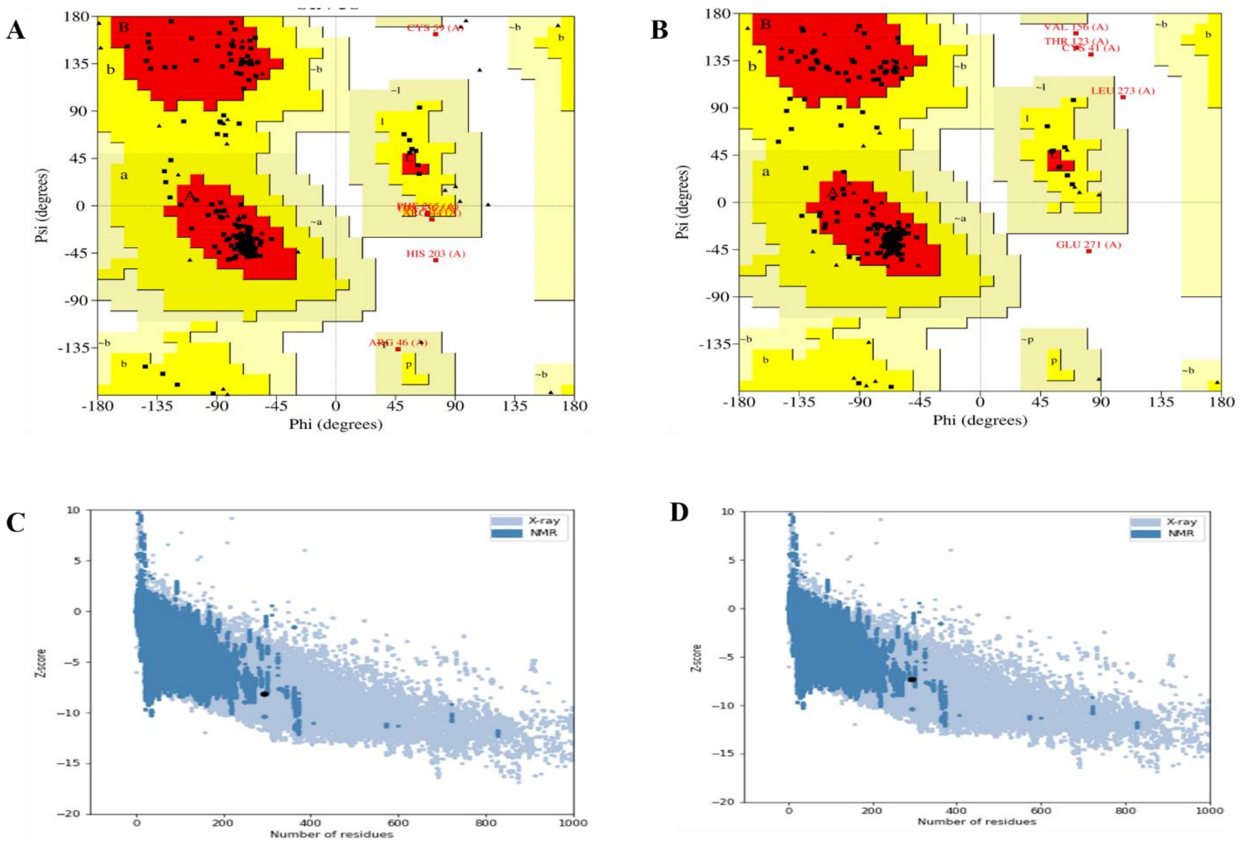

Figure S3: Three-dimensional structural validation of V1 and V3. (A) (B) Ramachandran plots showing 89.1% residues of V1 and 89.96% residues of V3 in the most favored regions of plots. (C) (D) structural validation by proSA web showing Z scores of -8.11 and -7.28 of V1 and V3 respectively.

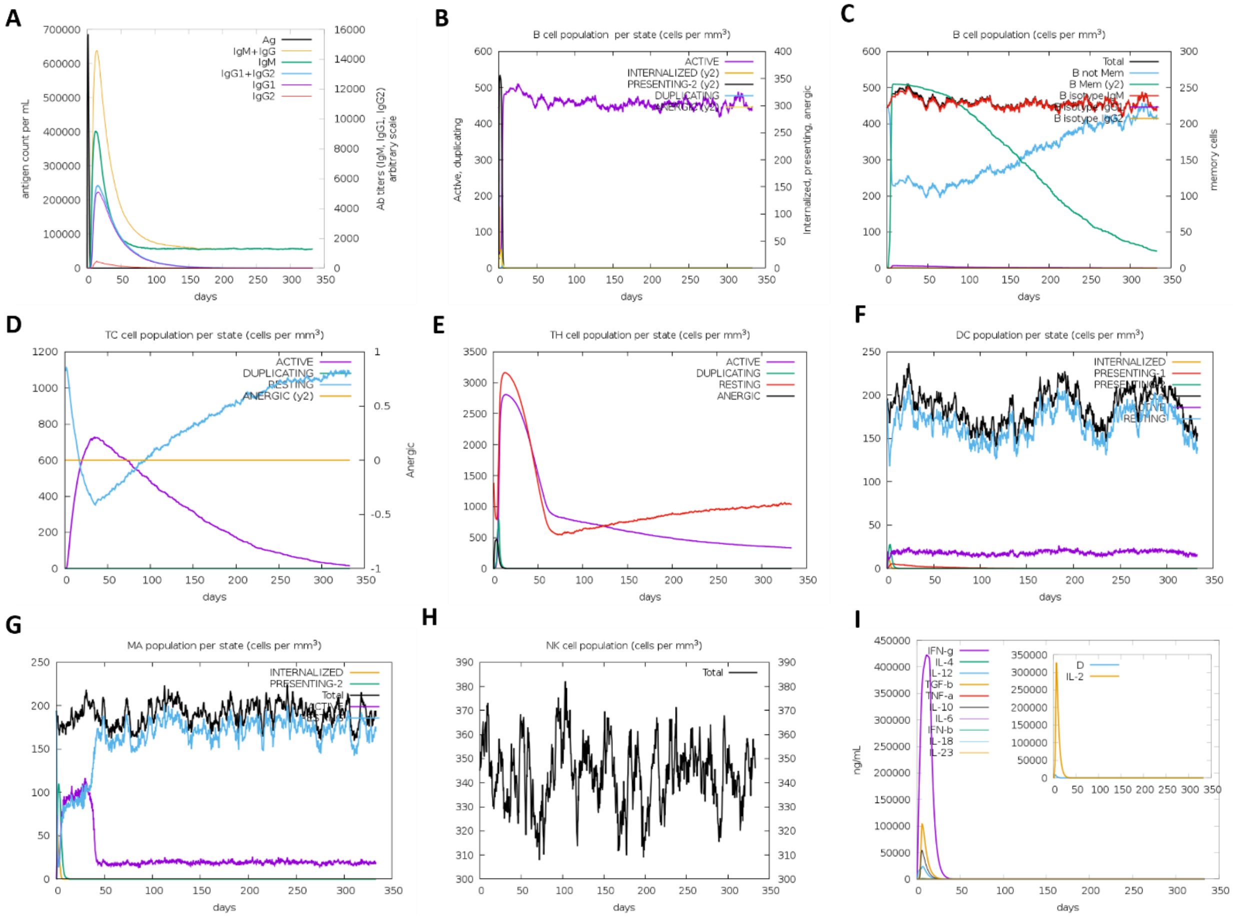

Figure S4**:** *In silico* immune simulation of LSDV-V2 chimeric peptide predicted by C-ImmSim Server. (A, B) Elevated level of immunoglobin antibodies as well as B – cell populations while a decrease in antigen levels upon vaccine injections. (C) Significant increase in B- cell populations after repeated exposure to antigen. (D, E) The increase in the population of T -cytotoxic and T –helper cells upon repeated exposure. (F, G, H) The population increase of dendritic cells, macrophages, and natural killer cells during the immunization period. (I) increased concentration of cytokine and interleukin levels after repeated antigen exposure. Inset plot shows danger signal together with leukocyte growth factor IL-2.

Section S1

mRNA Vaccine Construct

5′ m7GCap– 5′ UTR–Kozak sequence–Signal peptide (tPA)–EAAAK-Adjuvant-peptide vaccine sequence-MITD sequence- Stop codon- 3′ UTR-Poly (A) tail.

5'm7G Cap

5'UTR
NCA-7d (Derived from NCA-7)
GGCAAAAAUCAAAAUCAAUCAUCAUCACAACAUCAACAAUCAAUCAUCAACACAUCAUCAAGACACCA
CCGCCGCCGCCAUGGCG

Kozak Sequence
GCCGCC(A/G)(C/A)CAUGGCG
GCCGCCGCCAUGGCG

Signal Peptide
>sp|Q28198|TPA_BOVIN Tissue-type plasminogen activator OS=Bos taurus
ATGATGAGCGCCATGAAGACCGAGTTCCTGTGCGTGCTGCTGCTGTGCGGCGCCGTGTTCACCAGCCCCAGC
CAGGAGACCTACAGGAGGCTGAGGAGGGGCGCCAGGAGCTACAAGGTGACCTGCAGGGACGGCAAGACCC
AGATGACCTACAGGCAGCACGACAGCTGGCTGAGGCCCCTGCTGAGGGGCAACCAGGTGGAGCACTGCTGG
TGCGACGGCGGCAGGGCCCAGTGCCACAGCGTGCCCGTGAGGAGCTGCAGCGAGCCCTGGTGCTTCAACGG
CGGCACCTGCAGGCAGGCCCTGTACAGCAGCGACTTCGTGTGCCAGTGCCCCGAGGGCTTCATGGGCAAGC
TGTGCGAGATCGACGCCACCGCCACCTGCTACAAGGACCAGGGCGTGGCCTACAGGGGCACCTGGAGCACC
GCCGAGAGCGGCGCCGAGTGCGCCAACTGGAACAGCAGCGGCCTGGCCATGAAGCCCTACAGCGGCAGGA
GGCCCAACGCCATCAGGCTGGGCCTGGGCAACCACAACTACTGCAGGAACCCCGACCAGGACAGCAAGCC
CTGGTGCTACGTGTTCAAGGCCGGCAAGTACATCAGCGAGTTCTGCAGCACCCCCGCCTGCGCCAAGGTGGC
CGAGGAGGACGGCGACTGCTACACCGGCAACGGCCTGGCCTACAGGGGCACCAGGAGCCACACCAAGAGC
GGCGCCAGCTGCCTGCCCTGGAACAGCGTGTTCCTGACCAGCAAGATCTACACCGCCTGGAAGAGCAACGC
CCCCGCCCTGGGCCTGGGCAAGCACAACCACTGCAGGAACCCCGACGGCGACGCCCAGCCCTGGTGCCACG
TGTGGAAGGACAGGCAGCTGACCTGGGAGTACTGCGACGTGCCCCAGTGCGTGACCTGCGGCCTGAGGCAG
TACAAGAGGCCCCAGTTCAGGATCAAGGGCGGCCTGTTCGCCGACATCACCAGCCACCCCTGGCAGGCCGC
CATCTTCGTGAAGAACAGGAGGAGCCCCGGCGAGAGGTTCCTGTGCGGCGGCATCCTGATCAGCAGCTGCT
GGGTGCTGAGCGCCGCCCACTGCTTCCAGGAGAGGTACCCCCCCCACCACCTGAAGGTGTTCCTGGGCAGG
ACCTACAGGCTGGTGCCCGGCGAGGAGGAGCAGACCTTCGAGGTGGAGAAGTACATCATCCACAAGGAGTT
CGACGACGACACCTACGACAACGACATCGCCCTGCTGCACCTGAAGAGCGACAGCCTGACCTGCGCCAGGG
AGAGCGCCAGCGTGAGGACCATCTGCCTGCCCGACGCCAGCCTGCAGCTGCCCGACTGGACCGAGTGCGAG
CTGAGCGGCTACGGCAAGCACGAGAGCAGCAGCCCCTTCTTCAGCGAGAGGCTGAAGGAGGCCCACGTGA
GGCTGTACCCCAGCAGCAGGTGCACCAGCCAGCACCTGTTCAACAGGACCGTGACCAACAACATGCTGTGC
GCCGGCGACACCAGGAGCGGCGGCGACCACACCAACCTGCACGACGCCTGCCAGGGCGACAGCGGCGGCC
CCCTGGTGTGCATGAAGGACAACCACATGACCCTGGTGGGCATCATCAGCTGGGGCCTGGGCTGCGGCAGG
AAGGACGTGCCCGGCGTGTACACCAAGGTGACCAACTACCTGGACTGGATCAGGGACAACACCAGGCCC

mRNA Sequence
GAAGCUGCUGCUAAACUGGCUCUGCUGUUCCUGGUUCUGUCUGCUGGUUCUGGUUUCACCCAGGGUGU
UCGUAACCACGUUACCUGCCGUAUCAACCGUGGUUUCUGCGUUCCGAUCCGUUGCCCGGGUCGUACCCG
UCAGAUCGGUACCUGCUUCGGUCCGCGUAUCAAAUGCUGCCGUUCUUGGGAAGCUGCUGCUAAAGCUA
AAUUCGUUGCUGCUUGGACCCUGAAAGCUGCUGCUGGUGGUGGUUCUAACUCUGUUAUCGGUACCAAC
UACGAACUGCUGUGCAUCAACACCAAAGGUCCGGGUCCGGGUAAAAAACUGAUCAUCAAAGAAGUUAA
ACGUAAAUACCUGUCUGCUGCUUACAACUCUACCAUCGCUCUGGGUAAAAACGGUGGUGGUUCUCACG
AAUACGGUGCUGAAGCUCUGGAACGUGCUGGUGGUGGUGGUUCUCUGUCUAACAUCAAAAAAUCUUCU
AAAGGUGACAUCAACGCUUGCUACGGUCCGGGUCCGGGUAAAAAAGUUUCUUACAUCAUCUGCGUUAA
ACGUCUGUACAACGCUGCUUACAACGUUUCUUGCAACUACGUUUCUUACGGUGGUGGUUCUCACGAAU
ACGGUGCUGAAGCUCUGGAACGUGCUGGUGGUGGUGGUUCUAAAGGUUGCAUCGUUGAAUUCGGUUCU
CAGGAAAAAGUUUGCGUUACCGGUCCGGGUCCGGGUAAAAAAGACCUGUCUCUGCUGAAACGUCGUAU
CCAGAAAGUUGCUGCUUACGACUUCUGGAUCAAAUUCAUCUCUAUCCACGAAUACGGUGCUGAAGCUC
UGGAACGUGCUGGUGCUAAAUUCGUUGCUGCUUGGACCCUGAAAGCUGCUGCUGGUGGUGGUUCU

3' UTR
S27a + R3U
UUGUGUAUGCGUUAAUAAAAAGAAGGAACUCGUAAAAACUCAAUGUAUUUCUGAGGAAGCGUGGUGC
AUAAUGCCACGCAGCGUCUGCAUAACUUUUAUUAUUUCUUUUAUUAAUCAACAAA

Poly-A tail
AAAAAAAAAAAAAAAAAAAAAAAAAAAAAAAAAAAAAAAAAAAAAAAAAAAAAAAAAAAAAAAAAA
AAAAAAAAAAAAAAAAAAAAAAAAAAAAAAAAAAAAAAAAAAAAAAAAAAAAA
